# Early-Life Rotenone Exposure Enhances Nigrostriatal Vulnerability and Parkinsonian Neurodegeneration in Aging Rats

**DOI:** 10.3390/toxics14060470

**Published:** 2026-05-27

**Authors:** Margarita Gómez-Chavarín, Rocio Morales-Gómez, Juan Ramón Padilla-Mendoza, Patricia Padilla, Ismael Torres-Saldaña, Patricia Vergara-Aragón, Maria-del-Carmen Silva-Lucero, Nuria Galindo-Solano

**Affiliations:** 1 Departamento de Fisiología, Facultad de Medicina, Universidad Nacional Autónoma de México (UNAM), Av. Universidad 3000, Coyoacán, Ciudad de México C.P. 04510, Mexico; ibtramon.padilla@outlook.mx (J.R.P.-M.); pvergara@unam.mx (P.V.-A.); carmenaguila10@hotmail.com (M.-d.-C.S.-L.); 2 Hospital de la Mujer, Secretaría de Salud, Ciudad de México C.P. 11430, Mexico; drarociomorales@gmail.com; 3 Unidad de Cromatografía Líquida, Instituto de Investigaciones Biomédicas, Universidad Nacional Autónoma de México (UNAM), Av. Universidad 3000, Coyoacán, Ciudad de México C.P. 04510, Mexico; ppadillac@iibiomedicas.unam.mx; 4 Unidad Académica Bioterio, Facultad de Medicina, Universidad Nacional Autónoma de México (UNAM), Av. Universidad 3000, Coyoacán, Ciudad de México C.P. 04510, Mexico; ismael.torresdiaz@hotmail.com; 5 Facultad de Ciencias, Universidad Nacional Autónoma de México (UNAM), Av. Universidad 3000, Coyoacán, Ciudad de México C.P. 04510, Mexico; nuria.gs@ciencias.unam.mx

**Keywords:** rotenone, neurodevelopment, medium spiny neurons, dopaminergic neurons, senescence, Parkinson’s disease

## Abstract

Environmental exposure to neurotoxicants during critical developmental windows may program long-term susceptibility to neurodegenerative diseases such as Parkinson’s disease. Here, we investigated whether rotenone exposure during neurodevelopment induces a more severe Parkinsonian phenotype during aging than adult-onset exposure. Wistar rats were exposed to rotenone (1 mg/kg/day) either during gestation and lactation or from postnatal day 60 to 102. Motor performance was assessed longitudinally, and neurobiological analyses were conducted at 12 months of age. Developmental rotenone exposure induced persistent and severe motor deficits from early adulthood, whereas adult exposure resulted in a progressive phenotype. These alterations were accompanied by greater loss of tyrosine hydroxylase-positive dopaminergic neurons and a marked reduction in Nurr1 expression in the substantia nigra. Developmental exposure also increased cellular senescence, dendritic atrophy and spine loss in striatal medium spiny neurons, insoluble α-synuclein accumulation, and global DNA hypomethylation. Despite low residual serum rotenone levels, neurodegenerative alterations persisted, supporting a hit-and-run mechanism. These findings suggest that early-life rotenone exposure induces long-lasting epigenetic and cellular reprogramming that enhances nigrostriatal vulnerability and accelerates Parkinsonian neurodegeneration during aging.

## 1. Introduction

Parkinson’s disease (PD) is a progressive neurodegenerative disorder characterized by the selective loss of dopaminergic neurons in the substantia nigra and the consequent dysfunction of the nigrostriatal pathway. Although PD has traditionally been considered an age-related disease, increasing experimental and epidemiological evidence suggests that environmental factors, particularly exposure to neurotoxicants, may contribute to its pathogenesis. Among these factors, pesticides have been consistently associated with an increased risk of PD, highlighting the relevance of environmental exposures in PD pathogenesis.

Emerging evidence supports the concept that vulnerability to neurodegeneration may originate during early life. According to the developmental origins hypothesis, exposure to environmental insults during critical periods of brain development can induce long-lasting alterations that predispose individuals to neurological disorders later in life. In this context, early exposure to pesticides has been proposed to disrupt the maturation of the nigrostriatal dopaminergic system, thereby increasing its susceptibility to later neurodegenerative processes [1,2].

The nuclear receptor transcription factor Nurr1 (NR4A2) plays a central role in the development, differentiation, and maintenance of midbrain dopaminergic neurons. Nurr1 regulates the expression of key genes involved in dopamine synthesis, transport, and storage, including tyrosine hydroxylase (TH), dopamine transporter (DAT), and vesicular monoamine transporter 2 (VMAT2). In adulthood, Nurr1 is essential for maintaining dopaminergic neuron integrity, and its dysregulation has been implicated in the pathogenesis of PD [3]. Reduced Nurr1 expression has been associated with dopaminergic neuron loss, neuroinflammation, and altered cellular homeostasis [4,5].

In addition to transcriptional regulation, epigenetic mechanisms such as DNA methylation have emerged as critical regulators of gene expression in response to environmental exposures. Alterations in DNA methylation patterns may lead to persistent changes in neuronal function and survival. Environmental toxicants, including pesticides, have been shown to induce global DNA hypomethylation, potentially contributing to the dysregulation of genes involved in neuroprotection and synaptic plasticity. Furthermore, cellular senescence has been increasingly recognized as a contributor to neurodegenerative processes, particularly through the release of pro-inflammatory factors that exacerbate neuronal damage [4,6,7].

Rotenone (ROT), a lipophilic pesticide and mitochondrial complex I inhibitor, is widely used to model Parkinson’s disease because of its ability to reproduce key pathological features, including dopaminergic neurodegeneration, oxidative stress, and α-synuclein aggregation [8,9]. Although the effects of rotenone exposure in adult animals have been extensively studied, its impact during neurodevelopment and its long-term consequences for aging-related neurodegeneration remain incompletely understood. Previous work from our group demonstrated that early-life rotenone exposure induces dopaminergic alterations and Parkinsonian-like features in young adult rats. However, whether these early-life changes persist and exacerbate neurodegeneration during aging has not been fully investigated [10,11,12,13].

Epigenetic mechanisms, including DNA hypomethylation at CpG sites, may be critical in mediating the long-term effects of rotenone exposure during neurodevelopment [2]. These alterations may reduce Nurr1 protein expression, a factor essential for dopaminergic neuron differentiation and maintenance. Furthermore, the contribution of cellular senescence to this long-term vulnerability has not been fully elucidated. Therefore, this study aimed to determine whether rotenone exposure during neurodevelopment programs persistent susceptibility of the nigrostriatal dopaminergic system, leading to accelerated neurodegeneration during aging. We hypothesized that early-life exposure induces long-lasting epigenetic and cellular alterations that promote a more severe Parkinsonian phenotype later in life.

## 2. Materials and Methods

### 2.1. Animals and Experimental Design

**Figure 1:**
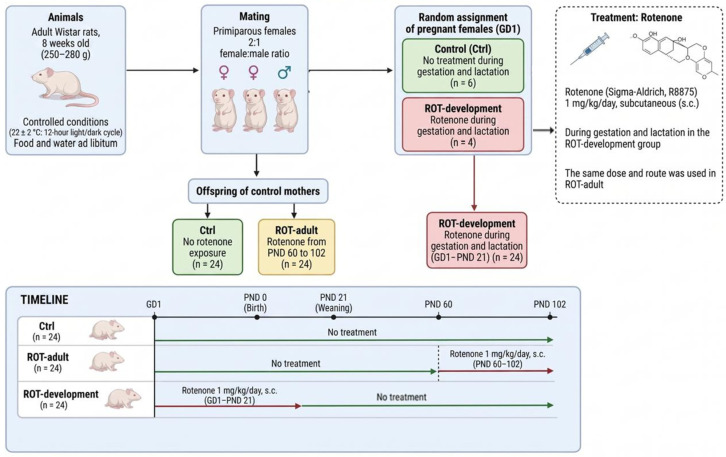
Experimental design and treatment timeline. Schematic representation of experimental groups and rotenone exposure periods during development and adulthood. GD1, gestational day 1; PND, postnatal day. Green indicates the untreated control group (Ctrl), whereas red indicates the rotenone-treated groups (ROT-adult and ROT-development).

Eight-week-old adult Wistar rats weighing 250–280 g were obtained from the Unidad Académica Bioterio de la Facultad de Medicina, Universidad Nacional Autónoma de México (UNAM). Animals were housed under controlled environmental conditions (22 ± 2 °C; 12 h light/dark cycle) with ad libitum access to food and water. Primiparous females were mated at a 2:1 female-to-male ratio, and pregnancy was confirmed by vaginal smear examination, which was considered gestational day 1 (Figure 1). Pregnant dams were randomly assigned to one of two groups:

- Control (Ctrl, n = 6), which received no treatment during gestation and lactation; and
- ROT-development (n = 4), which received rotenone during gestation and lactation.

At birth, offspring from control dams were further subdivided into two groups:

- Ctrl group, with no rotenone exposure; and
- ROT-adult group, which received rotenone from postnatal day (PND) 60 to 102.

Thus, three experimental groups were established:

- Ctrl (n = 24);
- ROT-adult (PND 60-102; n = 24); and
- ROT-development (gestation and lactation; n = 24).

Rotenone (Sigma-Aldrich, St. Louis, MO, USA; R8875) was administered subcutaneously at a dose of 1 mg/kg/day for 42 consecutive days, as previously described [2]. The dose and route of administration were selected based on previous studies showing reliable induction of dopaminergic alterations with minimal mortality. All experimental procedures were conducted in accordance with NOM-062-ZOO-1999 and institutional guidelines for the care and use of laboratory animals. Animal use complied with the ethical standards in place at the time the experiments were performed and with current institutional regulations (Project No. 033-607-17). Sample size was determined based on previous studies and the variability observed in similar experimental models.

### 2.2. Motor Coordination Assessment

Motor performance was evaluated in all experimental groups (n = 10 per group) at 2, 6, and 12 months of age using the beam-walking test [14]. All rats underwent a five-day training period between 09:00 and 11:00 h, during which they were trained to traverse a wooden beam measuring 2 m in length and 9 mm in width, inclined at 15°. During training, animals were placed at the lower end of the beam, and the time required to reach the home cage at the opposite end was recorded as latency and expressed in seconds. On the sixth day, the test was conducted under the same environmental and time conditions. For the evaluation, a narrower beam, 3 mm in width, was used, and the latency to complete the task was recorded. A cutoff time of 120 s was established. Rats that failed to cross the beam within this period were gently removed and assigned a latency score of 120 s.

### 2.3. Immunohistochemistry for Tyrosine Hydroxylase (TH-IR) and Nurr1 (Nurr1-IR)

At 12 months of age, rats from each experimental group (n = 4 per group) were deeply anesthetized with sodium pentobarbital (150 mg/kg, i.p.; Pfizer, Mexico City, Mexico) and transcardially perfused with 250 mL of phosphate-buffered saline (PBS; 0.1 M, pH 7.4), followed by 250 mL of 4% paraformaldehyde in PBS. Brains were then removed, postfixed overnight in the same fixative, and cryoprotected in graded sucrose solutions (15% and 30%) at 4 °C. Serial coronal sections, 40 µm thick, encompassing the entire substantia nigra were obtained using a freezing microtome (Cryo-Cut, American Optical, Burlington, ON, Canada) and collected in 24-well plates containing PBS.

Immunoperoxidase staining for tyrosine hydroxylase (TH; sc-14007, Santa Cruz Biotechnology, Dallas, TX, USA) and nuclear receptor-related 1 protein (Nurr1; ab227260, Abcam, MA, USA) was performed following established protocols [2]. TH-immunoreactive (TH-IR) neurons were visualized using the NovaRED^®^ peroxidase substrate kit (Vector Laboratories, SK-4800, Burlingame, CA, USA), whereas Nurr1-immunoreactive (Nurr1-IR) nuclei were detected using a DAB substrate kit (Vector Laboratories, SK-4100), according to the manufacturer’s instructions. Sections were mounted onto gelatin-coated slides, dehydrated, and coverslipped with Cytoseal 60 mounting medium (Thermo Fisher Scientific, Waltham, MA, USA; Cat. No. 8310-4).

### 2.4. Acquisition and Quantification of TH-IR Dopaminergic Neurons and Nurr1-IR Nuclei in the Substantia Nigra

Quantification was performed on six coronal sections per animal (n = 4 per group), selected at matched rostrocaudal levels of the substantia nigra and spaced 240 μm apart. Sections were imaged using a CCD camera (Reichert-Jung, New York, NY, USA) under consistent brightness and contrast settings at 40× for TH and 100× for Nurr1. Using Stereo Investigator software 2023.2.1.

TH-IR neurons with a soma diameter of 12–20 μm and clearly defined Nurr1-IR nuclei were manually counted within the substantia nigra in both hemispheres, using a counting frame area of 0.075 mm^2^ per section. The observers performing the counts were blinded to the experimental groups.

### 2.5. Histochemistry for Senescence-Associated β-Galactosidase (SA-β-gal)

A subset of midbrain sections (n = 4 animals per group; 6 sections per animal) was processed for senescence-associated β-galactosidase (SA-β-gal) histochemistry. Cryosections were incubated for 16 h at 37.5 °C in SA-β-gal staining solution (pH 6.0) containing 1 mg/mL X-gal (Invitrogen, Waltham, MA, USA; B-1690), and sections were processed as described by Debacq-Chainiaux et al. [15]. After incubation, sections were washed, briefly counterstained with nuclear fast red, dehydrated, and mounted with VectaMount (Vector, Newark, CA, USA; H-5502). SA-β-gal-positive cells were identified by the presence of a blue-green precipitate.

### 2.6. Dendritic Length and Spine Density of Striatal Medium Spiny Neurons

Four 12-month-old rats per group were deeply anesthetized with sodium pentobarbital (18.5 mg/kg, i.p.; Pfizer, Mexico City, Mexico) and euthanized by decapitation. Brains were rapidly removed and processed using the FD Rapid GolgiStain™ Kit (NeuroTechnologies, Columbia, MD, USA; PK-401). Each brain was carefully extracted to avoid tissue damage and briefly rinsed in double-distilled water to remove surface blood.

For impregnation, equal volumes of Solutions A and B were mixed 24 h in advance. Brains were immersed in this solution for 2 weeks at room temperature in the dark, followed by transfer to Solution C for an additional week under the same conditions. Coronal sections of the dorsal striatum (100 µm thick) were obtained using a vibratome (Leica; Buffalo Grove, IL, USA; VT1000S) and collected in Solution C at 4 °C. Sections were mounted onto gelatin-coated slides and stained using a freshly prepared solution containing 1 part Solution D, 1 part Solution E, and 2 parts distilled water.

After staining, excess solution was removed, and sections were dehydrated through a graded ethanol series: 50%, 75%, and 90% ethanol for 30–50 s each, followed by 100% ethanol. Sections were then cleared in xylene and coverslipped with Cytoseal 60 mounting medium (Thermo Fisher, Cat. No. 8310-4). Medium spiny neurons (MSNs) in the dorsal striatum were analyzed using Sholl analysis [16]. Dendritic arborization was assessed by counting the number of intersections, and total dendritic length was measured in micrometers. Spine density was quantified as the number of spines per 10 µm segment along dendritic branches.

Neurons were selected under 100× magnification using an Olympus BX51 light microscope (Olympus, Melville, NY, USA). Only well-impregnated neurons with intact dendritic fields and no staining artifacts, blood vessels, or precipitates were included. Ten neurons per hemisphere from six sections per animal were imaged using a CMOS camera (Hamamatsu Photonics Hamamatsu City, Shizuoka, Japan).

### 2.7. Estimation of Insoluble α-Synuclein Concentration in the Striatum

To quantify insoluble α-synuclein (α-synif) content in the striatum, 12-month-old rotenone-exposed rats (n = 5 per group) were deeply anesthetized with sodium pentobarbital (18.5 mg/kg, i.p.; Pfizer, Mexico City, Mexico) and euthanized by decapitation. Brains were rapidly removed, and striatal tissue samples were processed as described by Campbell et al. [17].

Briefly, striatal tissue was homogenized by sonication at 4 °C in TBS buffer containing protease inhibitors at a 1:10 ratio (*w*/*v*): 50 mM Tris-HCl, 175 mM NaCl, pH 7.4, 2 mM PMSF, 5 mM EDTA, 2 µg/mL aprotinin, 2 µg/mL pepstatin A, 5 µg/mL leupeptin, and 2 µg/mL antipain. The homogenate was centrifuged at 1000× *g* for 5 min at 4 °C, and the resulting supernatant was collected as the crude α-synuclein homogenate. This homogenate was then centrifuged at 100,000× *g* for 60 min at 4 °C to obtain the TBS-soluble α-synuclein fraction.

The pellet was washed twice with TBS and resuspended by sonication at room temperature in TBS buffer containing 5% SDS. The suspension was centrifuged at 100,000× *g* for 30 min at 25 °C, and the resulting supernatant was collected as the SDS-soluble α-synuclein fraction. Finally, the pellet was washed twice with TBS buffer and resuspended in 8 M urea and 8% SDS to obtain the insoluble α-synuclein fraction.

The concentration of α-synif was estimated using the Biotrak ELISA system, according to the manufacturer’s instructions (Amersham Biosciences, Little Chalfont, Buckinghamshire, UK; RPN5902) The ELISA assay was performed in triplicate by adding 0.1 mg of protein in 100 µL per well. The intra-assay coefficient of variation was <5%. A standard curve was generated using linear regression [2].

### 2.8. DNA Extraction and Global DNA Methylation Analysis

Genomic DNA was extracted from striatal tissue of 12-month-old rats from the Ctrl, ROT-adult, and ROT-development groups using the QIAamp DNA Mini Kit (Qiagen, 3 Hilden, Germany; 30Cat. No. 56304), according to the manufacturer’s instructions. DNA concentration and purity were assessed using a NanoDrop spectrophotometer (NanoDrop NS-1000, Thermo Fisher Scientific). Only samples with A260/A280 ratios of approximately 1.8 were included for further analysis.

For global DNA methylation analysis, striatal tissue samples (n = 4 per group) were carefully dissected under a stereomicroscope and stored at −70 °C until processing. Global 5-methylcytosine (5-mC) levels were quantified using a colorimetric ELISA-based Methylated DNA Quantification Kit (Epigentek, P1030-96, New York, NY, USA), according to the manufacturer’s protocol. Briefly, 100 ng of DNA per sample was used, and absorbance was measured at 450 nm using a microplate reader (BioTek Instruments Winooski, VT, USA). The percentage of global DNA methylation (%5-mC) was calculated using a standard curve generated by linear regression. The amount and percentage of 5-mC were calculated using the following equations:

5-mC (ng) = (Sample OD − Blank OD)/(Slope × 2)

5-mC (%) = [5-mC (ng)/total input DNA (ng)] × 100

### 2.9. Estimation of Rotenone Concentration in Serum

Rotenone concentration was determined in blood samples collected from six 12-month-old rats per group. A total of 200 µL of serum was processed for HPLC analysis, as previously reported [2]. Samples were centrifuged at 3000 rpm for 10 min at room temperature to separate the serum, which was then stored at −70 °C until instrumental analysis.

Before HPLC analysis, serum samples were precipitated with acetonitrile (*v*/*v*), and the resulting supernatant was collected by centrifugation and evaporated under vacuum. The precipitate was reconstituted with 30 µL of HPLC-grade acetonitrile, and 10 µL was injected into the system.

A Waters HPLC-UV system equipped with a SunFire C18 column (5 µm particle size, 4.6 mm × 150 mm; Cat. No. 186002559; Milford, MA, USA) was used for chromatographic separation. The mobile phase consisted of a gradient of water and 100% acetonitrile. The gradient started with 10% aqueous phase (A) and 90% acetonitrile (B), shifted to 70% A and 30% B after 2 min, and reached 75% A and 25% B after 12 min. The flow rate was maintained at 1.0 mL/min at room temperature. Rotenone was detected by measuring UV absorbance at 290 nm.

Detection and quantification of rotenone were performed using Empower^®^ software (Version 71500031203; Milford, MA, USA). The retention time of rotenone was 7.35 min. The method showed a limit of detection (LOD) of 0.12 µg/mL and a limit of quantification (LOQ) of 0.35 µg/mL. The method demonstrated linearity (r^2^ > 0.999) within a quantification range of 0.25–250 µg/mL, recovery of 94-112%, selectivity, with no endogenous or exogenous interferences detected, and reproducibility (CV < 7.81%) [2].

### 2.10. Statistical Analysis

All cell counts were performed by observers blinded to the experimental groups. Data are presented as the mean ± standard error of the mean (SEM). Differences in latency (s), TH-immunoreactive (TH-IR) neuron number, Nurr1-immunoreactive (Nurr1-IR) nuclei number, percentage of global DNA methylation (5-mC), and dendritic spine density of medium spiny neurons (MSNs) in the striatum were analyzed using one-way analysis of variance (ANOVA), followed by Tukey’s post hoc test.

In contrast, α-synuclein levels and dendritic length of MSNs in the striatum were analyzed using the Kruskal-Wallis test followed by Dunn’s multiple comparisons test. Serum rotenone concentrations were evaluated using an unpaired *t*-test with Welch’s correction. Statistical significance was set at *p* < 0.05 (α = 0.05).

Pearson’s correlation analysis was used to assess the relationships between TH-IR neuron number and Nurr1-IR nuclei number in the substantia nigra, as well as between dendritic length and spine density in the striatum. All statistical analyses and graphical representations were performed using GraphPad Prism version 10.1.1 (GraphPad Software, La Jolla, CA, USA).

## 3. Results

### 3.1. Developmental Rotenone Exposure Induces Persistent Motor Impairment

**Figure 2:**
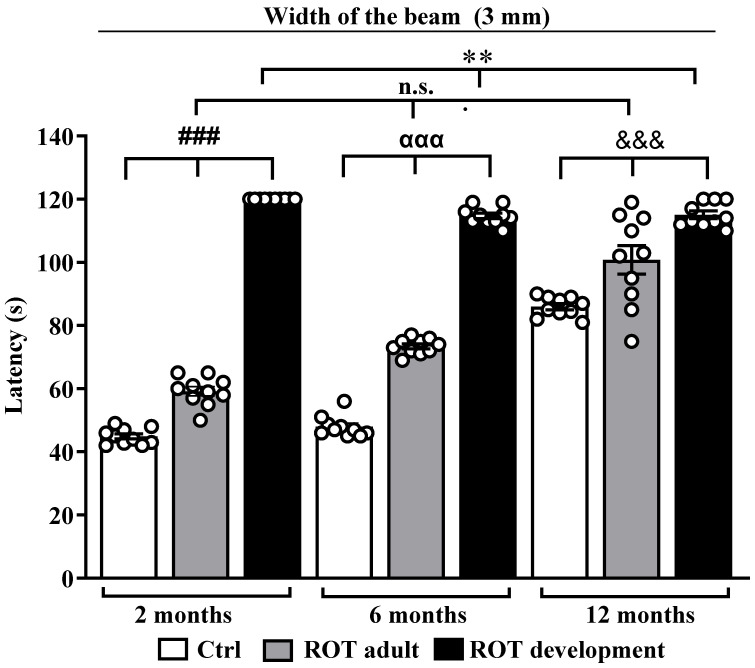
Motor coordination assessed by the beam-walking test using a 3 mm-wide beam inclined at 15°. Latency, expressed in seconds, was recorded at 2, 6, and 12 months of age. Control rats showed stable, short latencies across all ages. ROT-development rats exhibited maximal latency of 120 s at all time points, indicating an early, severe motor deficit that persisted over time. ROT-adult rats displayed progressive worsening, with significantly increased latency at 12 months. Data are presented as the mean ± SEM (n = 10 per group). ** *p* < 0.005; ###, ααα, &&& < 0.0001. All comparisons are relative to the Ctrl group at the corresponding time point.

Motor coordination was assessed using the beam-walking test at 2, 6, and 12 months of age (Figure 2). Rats exposed to rotenone during development showed severe and persistent motor deficits, reaching the maximal latency of 120 s at all evaluated time points. In contrast, rats exposed during adulthood displayed progressive motor impairment, with significant differences from control animals observed only at 12 months.

Statistical analysis revealed significant differences among groups (one-way ANOVA, *p* < 0.0001). Post hoc comparisons confirmed that the ROT-development group exhibited significantly greater impairment than both the Ctrl and ROT-adult groups. These findings indicate that early-life rotenone exposure induces long-lasting motor dysfunction, whereas adult exposure resulted in a progressive motor phenotype.

### 3.2. Developmental Rotenone Exposure Enhances Dopaminergic Neuron Loss and Reduces Nurr1 Expression

**Figure 3:**
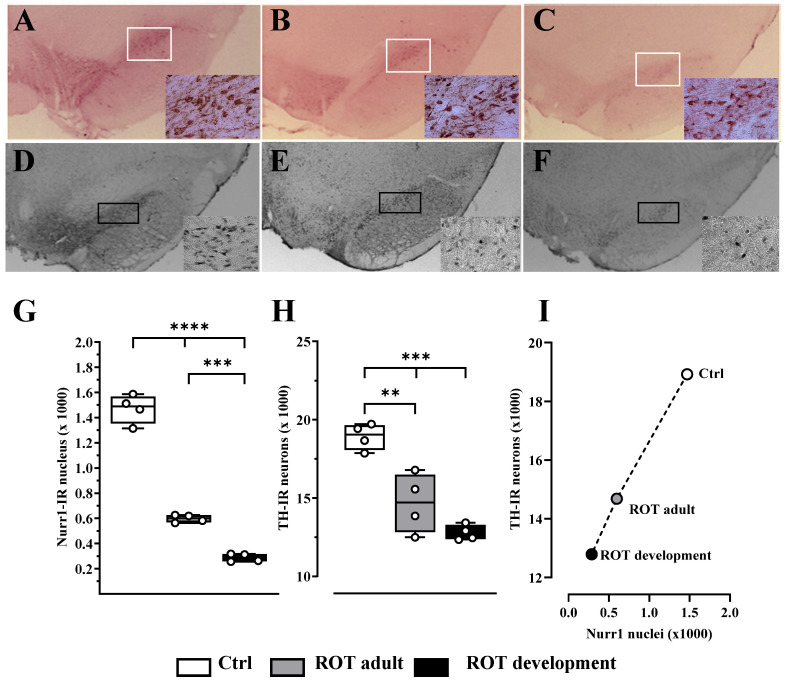
Immunohistochemical analysis of dopaminergic neurons in the substantia nigra at 12 months of age. (**A**–**C**) Representative photomicrographs of TH-immunoreactive (TH-IR) neurons in the substantia nigra. Control rats showed abundant, darkly stained dopaminergic neurons with extensive processes (**A**), whereas ROT-adult (**B**) and ROT-development (**C**) rats showed neuronal loss, with the most severe reduction and atrophic morphology observed in the ROT-development group. (**D**–**F**) Representative photomicrographs of Nurr1-immunoreactive (Nurr1-IR) nuclei in the same experimental groups. A marked reduction in immunoreactive nuclei was observed in rotenone-exposed animals, particularly in the ROT-development group. (**G**,**H**) Quantitative analysis confirmed significant loss of TH-IR neurons (**G**) and Nurr1-IR nuclei (**H**) in both rotenone-treated groups compared with control rats. Data are presented as the mean ± SEM (n = 4 per group). ** *p* < 0.05; *** *p* < 0.001; **** *p* < 0.0001. (**I**) Pearson’s linear correlation between TH-IR neuron number and Nurr1-IR nuclei number revealed a strong positive association (r^2^ = 0.9975; *p* = 0.0319). Scale bars = 50 µm.

Immunohistochemical analysis of the substantia nigra at 12 months revealed a marked reduction in TH-positive dopaminergic neurons in both rotenone-exposed groups, with the most pronounced effect observed in the ROT-development group (Figure 3A–C). Surviving neurons displayed reduced soma size, weaker immunoreactivity, and shortened neuronal processes compared with controls.

Similarly, Nurr1-immunoreactive nuclei were significantly decreased in rotenone-treated animals, with the lowest levels observed in the ROT-development group (Figure 3D–F). Quantitative analysis confirmed significant differences in TH-positive neuron number (F(2,9) = 26.39, *p* < 0.0001) and Nurr1-positive nuclei number (F(2,9) = 298.3, *p* < 0.0001). A strong positive correlation was observed between TH-positive neuron number and Nurr1-positive nuclei number (r^2^ = 0.9975, *p* = 0.0319; Figure 2), suggesting a close association between Nurr1 expression and dopaminergic neuron survival.

### 3.3. Developmental Rotenone Exposure Increases Cellular Senescence in the Substantia Nigra

**Figure 4:**
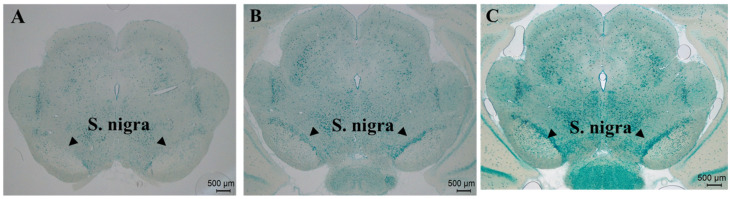
Representative low-magnification micrographs of SA-β-gal histochemistry in the substantia nigra. (**A**) Control, (**B**) ROT-adult, and (**C**) ROT-development groups. Increased SA-β-gal activity, identified by dark blue-green precipitate, was observed in rotenone-exposed animals and was most pronounced in the ROT-development group, suggesting accelerated cellular senescence. Arrows indicate the substantia nigra. Magnification: 60×.

Representative substantia nigra sections, outlined by a dotted line, were stained for SA-β-gal histochemistry in aged rats from each experimental group and digitized using a Leica EZ4E microscope (Figure 4A–C). SA-β-gal-positive cells were identified by the presence of a blue-green precipitate in the substantia nigra.

In control rats, only occasionally faintly blue-green SA-β-gal-positive cells were observed (Figure 4A). In the ROT-adult group, a moderate increase in both the number and staining intensity of senescent cells was detected throughout the nigral region (Figure 4B). In contrast, the ROT-development group exhibited a dense accumulation of intensely stained SA-β-gal-positive cells distributed across the substantia nigra (Figure 4C), suggesting an accelerated and widespread cellular senescence phenotype.

Based on morphology, both neuron-like cells, characterized by large, rounded somata, and glial-like cells, characterized by smaller, irregular somata, were observed among the SA-β-gal-positive population. These findings suggest that developmental rotenone exposure accelerates cellular aging processes in the nigrostriatal system to a greater extent than adult exposure.

### 3.4. Developmental Rotenone Exposure Induces Structural Alterations in Medium Spiny Neurons and Increases Insoluble α-Synuclein Accumulation

**Figure 5:**
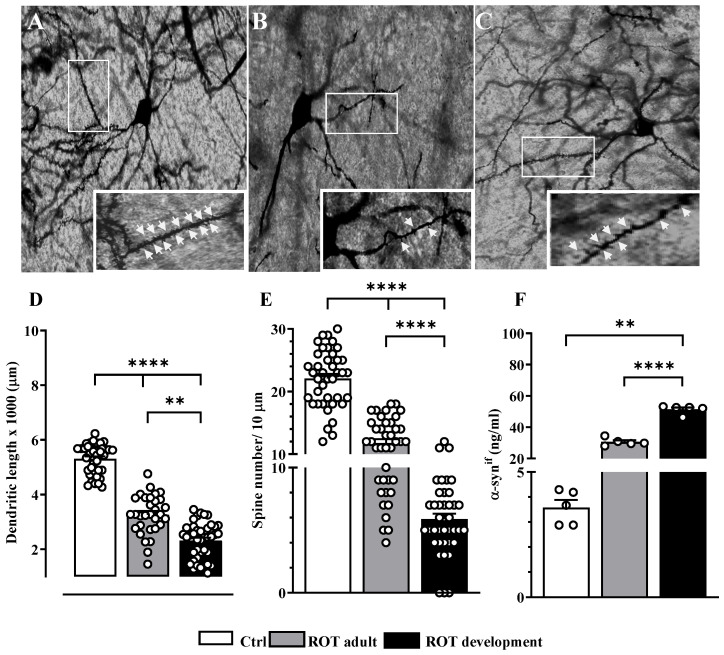
Morphological and biochemical alterations in the striatum of 12-month-old rats. (**A**–**C**) Representative photomicrographs of Golgi-impregnated secondary dendrites from medium spiny neurons (MSNs) in the dorsal striatum. Control rats exhibited abundant mature dendritic spines and preserved dendritic morphology (**A**), whereas ROT-adult rats showed a moderate reduction in spine density and dendritic complexity (**B**). In contrast, ROT-development rats displayed marked dendritic atrophy accompanied by a pronounced loss of dendritic spines (**C**). Insets show higher-magnification images of the selected dendritic segments (white boxes), and arrowheads indicate dendritic spines. Scale bar = 10 µm. (**D**,**E**) Quantitative analyses of dendritic length (**D**) and spine number per 10 µm (**E**) demonstrated significant dendritic degeneration and spine loss in rotenone-treated animals, with the most severe alterations observed in the ROT-development group. Data are presented as mean ± SEM (n = 4 animals per group). ** *p* < 0.01; **** *p* < 0.0001. (**F**) Insoluble α-synuclein (α-synif) levels in striatal homogenates determined by ELISA. Both rotenone-treated groups showed significantly increased α-synuclein accumulation compared with the control group, whereas the ROT-development group exhibited the highest levels. Data are presented as mean ± SEM (n = 5 animals per group). ** *p* < 0.01; **** *p* < 0.0001. Arrows indicate dendritic spines.

Dendritic length and spine density of medium spiny neurons (MSNs) in the striatum of 12-month-old control and rotenone-treated rats were analyzed using Golgi impregnation. Representative micrographs of striatal MSNs revealed a marked reduction in dendritic spine number and density in rotenone-exposed rats (Figure 5A–C). Control MSNs displayed a complex, highly branched dendritic arbor with a dense population of mature dendritic spines, many showing mushroom-like morphology (Figure 4A).

In the ROT-adult group, visible simplification of the dendritic tree was observed, accompanied by a moderate reduction in spine density, with many remaining spines appearing thin or filopodia-like (Figure 4B). The ROT-development group exhibited the most pronounced structural alterations, with MSNs showing markedly shortened dendritic branches, widespread spine loss, and an overall atrophic appearance (Figure 5C).

The Kruskal-Wallis test followed by Dunn’s multiple comparisons test revealed significant differences in dendritic length among groups (H = 84.94, *p* < 0.0001). One-way ANOVA followed by Tukey’s multiple comparisons test also revealed significant differences in spine density (F(2,117) = 183.3, *p* < 0.0001; r^2^ = 0.7581).

To further assess striatal pathology, the concentration of insoluble α-synuclein (α-synif) was estimated in the striatum. ELISA analysis revealed that insoluble α-synuclein concentration was significantly elevated in both rotenone-exposed groups. Notably, the ROT-development group exhibited a 14.5-fold increase over controls, whereas the ROT-adult group showed an 8.5-fold increase. The increase observed in the ROT-development group was significantly greater than that observed in the ROT-adult group, indicating that early-life rotenone exposure promotes a higher burden of pathological α-synuclein accumulation during aging than adult-onset exposure. The Kruskal-Wallis test followed by Dunn’s multiple comparisons test confirmed significant differences among groups (H = 12.52, *p* < 0.0001; Figure 5F).

### 3.5. Developmental Rotenone Exposure Induces Persistent Global DNA Hypomethylation

**Figure 6:**
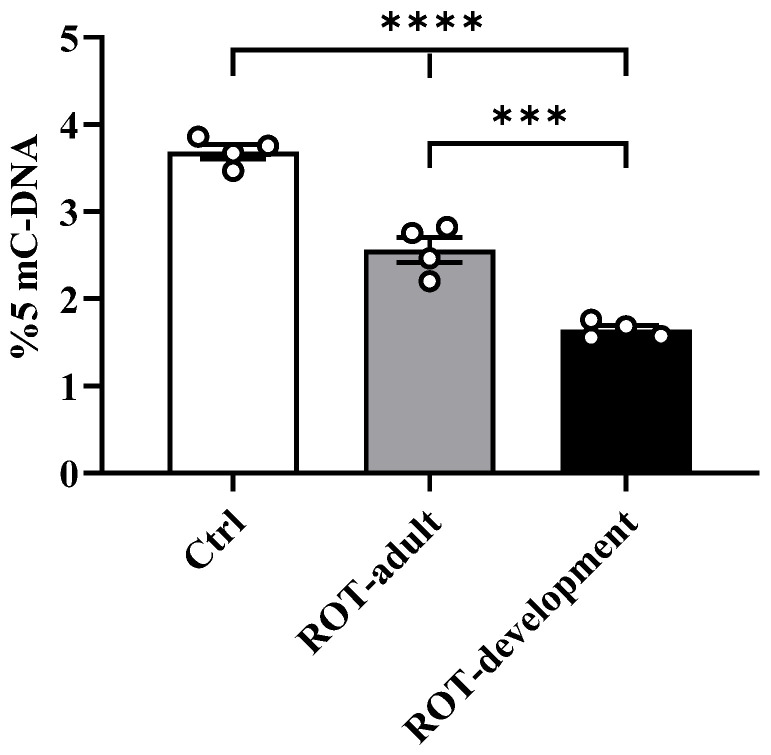
Global DNA methylation, expressed as 5-mC percentage, in the striatum at 12 months of age. Both rotenone-exposed groups exhibited significant hypomethylation compared with control rats. The ROT-development group showed the most pronounced reduction in 5-mC levels, consistent with persistent epigenetic reprogramming induced by early-life rotenone exposure. Data are presented as the mean ± SEM (n = 4 per group). *** *p* < 0.005; **** *p* < 0.0001.

Global DNA methylation analysis showed a significant reduction in 5-mC levels in the striatum of rotenone-exposed animals at 12 months of age (Figure 6). The ROT-development group exhibited the lowest methylation levels, which were significantly lower than those observed in both the Ctrl and ROT-adult groups (one-way ANOVA, F(2,9) = 105.7, *p* < 0.0001). These findings suggest that early-life rotenone exposure induces long-lasting epigenetic alterations consistent with persistent global DNA hypomethylation.

### 3.6. Low Residual Rotenone Levels Persist in Serum

**Figure 7:**
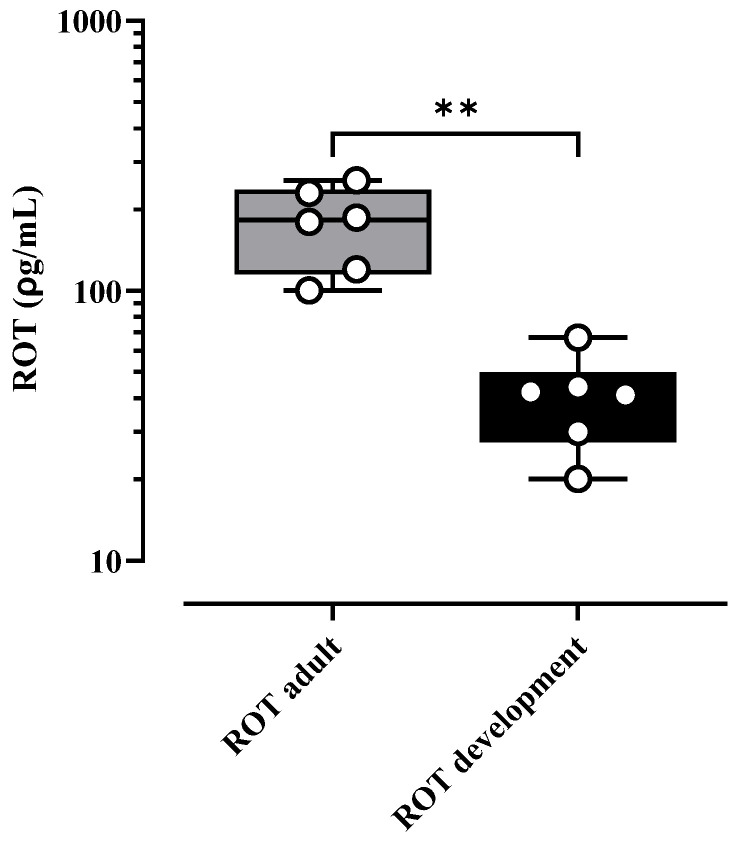
Rotenone concentration in serum at 12 months of age, determined by HPLC. Several months after the last administration, low residual rotenone levels were detected in both rotenone-exposed groups, with significantly higher concentrations in the ROT-adult group than in the ROT-development group. No rotenone was detected in control rats. Data are presented as the mean ± SEM (n = 6 per group). ** *p* = 0.0020, unpaired *t*-test with Welch’s correction.

HPLC analysis revealed low but detectable levels of rotenone in serum samples from rotenone-exposed animals at 12 months of age (Figure 7). The ROT-adult group showed significantly higher serum rotenone concentrations than the ROT-development group (*p* = 0.0020), whereas rotenone was not detected in control animals. Despite these low residual concentrations, the ROT-development group exhibited the most severe neurodegenerative phenotype, suggesting that the long-term effects of early-life exposure are not solely dependent on persistent toxin presence but rather on sustained molecular and cellular alterations.

## 4. Discussion

In the present study, we show that exposure to rotenone during neurodevelopment induces long-lasting alterations in the nigrostriatal dopaminergic system, leading to a more severe Parkinsonian phenotype during aging than exposure in adulthood. Our findings provide experimental evidence supporting the hypothesis that vulnerability to neurodegeneration may originate early in life and remain latent until it is exacerbated by aging-related processes.

A key finding was the distinct pattern of motor impairment observed between exposure periods. Rats exposed to rotenone during development exhibited persistent and severe deficits from early adulthood, whereas rats exposed during adulthood showed a progressive decline. This difference suggests that early-life exposure may interfere with the normal maturation of the dopaminergic system, leading to long-term functional impairment rather than a purely late-onset degenerative process. These observations are consistent with the concept that early-life environmental insults can program long-term neurological outcomes [18,19].

At the cellular level, developmental rotenone exposure resulted in greater loss of dopaminergic neurons in the substantia nigra, accompanied by a marked reduction in Nurr1 expression. Given the central role of Nurr1 in the development, maintenance, and survival of dopaminergic neurons, its downregulation may represent a mechanistic link between early exposure and later neurodegeneration. The strong positive correlation observed between Nurr1 expression and dopaminergic neuron number further supports the interpretation that transcriptional dysregulation contributes to neuronal vulnerability [20].

In addition to transcriptional alterations, we observed a significant increase in senescence-associated β-galactosidase activity in the substantia nigra, particularly in the ROT-development group. This finding suggests that early-life exposure accelerates cellular aging processes within the nigrostriatal system [21]. Cellular senescence is increasingly recognized as a contributor to neurodegeneration, not only due to irreversible cell cycle arrest but also through the secretion of pro-inflammatory and neurotoxic factors associated with the senescence-associated secretory phenotype (SASP). The accumulation of senescent cells may therefore create a pro-degenerative microenvironment that exacerbates neuronal damage over time.

Structural alterations in the striatum further support the presence of widespread neurobiological dysfunction. The reduction in dendritic length and spine density in medium spiny neurons reflects a loss of synaptic complexity and connectivity, which are essential for proper motor function. In parallel, the increased accumulation of insoluble α-synuclein is consistent with pathological aggregation processes characteristic of Parkinson’s disease. Together, these findings suggest that early-life rotenone exposure disrupts not only dopaminergic neurons but also the broader neuronal circuitry of the basal ganglia.

Epigenetic mechanisms, particularly DNA methylation, provide a plausible framework through which early environmental exposures can induce long-lasting biological effects [22,23]. In this study, the persistent global DNA hypomethylation observed in the ROT-development group provides a plausible mechanistic basis for an enduring neurodegenerative phenotype. Rotenone, as a mitochondrial complex I inhibitor, induces oxidative stress that may disrupt the activity of DNA methyltransferases (DNMTs) and ten-eleven translocation (TET) enzymes by altering levels of key metabolic cofactors such as S-adenosylmethionine and α-ketoglutarate [24,25]. This epigenetic imbalance may lead to aberrant gene expression affecting neuronal survival, synaptic function, and inflammatory responses.

Importantly, these epigenetic alterations may affect regulatory mechanisms involved in Nurr1 expression, providing a potential link between epigenetic dysregulation and the sustained downregulation of this transcription factor, which is essential for dopaminergic neuron maintenance [3,21]. This interpretation is consistent with the strong positive correlation observed between Nurr1 expression and dopaminergic neuron survival.

Together, these findings support a “hit-and-run” model, in which early toxic exposure leaves persistent epigenetic marks that shape long-term neuronal vulnerability. Notably, although neurodegenerative changes were evident at 12 months, only low levels of rotenone were detected in serum. The higher residual levels observed in the adult-exposed group may reflect rotenone sequestration in adipose tissue and gradual release over time [26]. In contrast, developmental exposure likely involves distinct pharmacokinetic dynamics due to maternal metabolism and lactational transfer.

However, the most relevant observation is that the ROT-development group, despite having the lowest residual toxin levels, exhibited the most severe neurodegenerative phenotype. This dissociation suggests that long-term effects are driven by persistent molecular and cellular alterations rather than ongoing toxic exposure, further supporting a “hit-and-run” mechanism. These findings extend our previous work by demonstrating that the effects of developmental exposure are not transient but persist and worsen with aging, ultimately leading to a more pronounced neurodegenerative phenotype.

Collectively, these data support a model in which early-life epigenetic and transcriptional dysregulation initiates a self-sustaining pathological cascade involving cellular senescence, neuroinflammation driven by the senescence-associated secretory phenotype, and progressive α-synuclein aggregation [10,11]. The convergence of these processes may establish a feed-forward loop of neurodegeneration that evolves over time, transforming an early-life insult into a clinically relevant Parkinsonian phenotype during aging. This framework reinforces the developmental origins hypothesis of Parkinson’s disease and highlights early-life environmental exposure as a critical determinant of long-term neurodegenerative risk. These findings may be particularly relevant in the context of human environmental exposure to mitochondrial toxicants, suggesting that early-life exposure could contribute to increased susceptibility to Parkinson’s disease later in life.

## 5. Conclusions

Early-life rotenone exposure induces long-lasting alterations that increase the vulnerability of the nigrostriatal dopaminergic system to neurodegeneration during aging. Developmental rotenone exposure results in more severe motor impairment, dopaminergic neuron loss, reduced Nurr1 expression, and increased cellular senescence compared with adult exposure. These alterations are accompanied by striatal structural damage, pathological α-synuclein accumulation, and persistent global DNA hypomethylation, suggesting sustained epigenetic and cellular reprogramming.

Importantly, these effects occur despite minimal residual toxin levels, supporting a “hit-and-run” mechanism. Collectively, our findings support the concept that early-life environmental exposure can program long-term neurodegenerative risk through interconnected epigenetic, transcriptional, and cellular aging mechanisms, reinforcing the developmental origins hypothesis of Parkinsonian neurodegeneration.

## Data Availability

The raw data supporting the conclusions of this study are available from the corresponding author upon reasonable request.
